# Clinical Application of Intravenous Lipid Emulsion Therapy in Cocaine-associated Cardiac Arrest: A Case Report

**DOI:** 10.5811/cpcem.50763

**Published:** 2026-05-23

**Authors:** Ryan Offman, Sarah K. Baribeau

**Affiliations:** * Trinity Health – Muskegon, Department of Emergency Medicine, Muskegon, Michigan; † Michigan State University College of Osteopathic Medicine, Department of Osteopathic Medical Specialties, East Lansing, Michigan

**Keywords:** cocaine, overdose, intravenous lipid emulsion, case report

## Abstract

**Introduction:**

Cardiac arrest in the setting of cocaine use portends high morbidity and mortality secondary to its powerful sodium channel blockade effects. Intravenous (IV) lipid emulsion has long been used as a rescue therapy in lipophilic toxicities.

**Case Report:**

We report a case in which IV lipid emulsion was used to successfully stabilize a patient who suffered cocaine-associated, out-of-hospital cardiac arrest.

**Conclusion:**

Intravenous lipid emulsion was used in the successful resuscitation of a cocaine overdose and could be considered for use in patients with cocaine-associated cardiac arrest.

## INTRODUCTION

Cocaine is a potent lipophilic sodium channel blocker and dopamine reuptake inhibitor. The resultant sodium blockade combined with an increase in circulating catecholamines can lead to life-threatening consequences, primarily dysrhythmias. This risk is further compounded by cocaine’s local anesthetic effects, which theoretically blunt impulse conduction and amplify the likelihood of dysrhythmias.^1^ Intravenous (IV) lipid emulsion exploits the lipophilic nature of various toxic substances by creating a lipid sink.^2^ While it has been researched in the context of anesthetic toxicity, IV lipid emulsion may theoretically be effective in cocaine toxicity via the same mechanism.

## CASE REPORT

A male in his early 30s with no significant past medical history presented to the emergency department (ED) after a witnessed cardiac arrest. Bystanders initiated cardiopulmonary resuscitation (CPR) after he exhibited an abrupt change in mental status and was found to be pulseless. Acute substance intoxication was suspected. Emergency medical services were summoned, and the patient was transported to the ED while the paramedics delivered compressions via a Lund University Cardiopulmonary Assist System. They had already administered 4 mg of IV naloxone and five 1-mg doses of IV epinephrine (0.1 mg per milliliter [mL]).

**Image 1:**
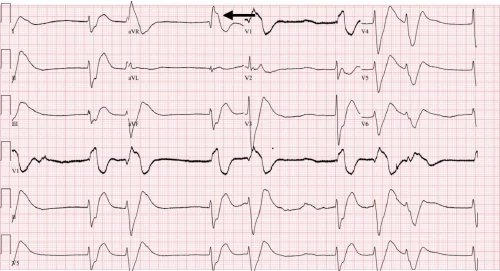
Initial electrocardiogram showing an irregular wide complex bradycardia at a rate of 50 beats per minute, QRS duration of 165 milliseconds, and prominent R wave in aVR (arrow).

Upon arrival, the patient’s initial cardiac rhythm was a wide complex pulseless electrical activity (PEA). He was intubated and received 50 milliequivalents (mEq) of IV sodium bicarbonate to address the wide complex PEA. A point-of-care basic metabolic panel lab revealed no significant electrolyte abnormalities. On the next pulse check, the patient achieved return of spontaneous circulation (ROSC). The immediate post-ROSC electrocardiogram (ECG) is shown below (Image 1).

Given the high suspicion of ingestion, he received an additional 4 mg of IV naloxone, and a nasogastric tube was placed for the administration of activated charcoal. The patient’s family arrived and confirmed that he had rapidly ingested a large quantity of cocaine mixed in water. Shortly thereafter, he lost pulses again, and CPR was resumed. He received an additional 50 mEq of IV sodium bicarbonate and 1 mg of IV epinephrine (0.1mg/mL), which resulted in a second ROSC.

**Image 2:**
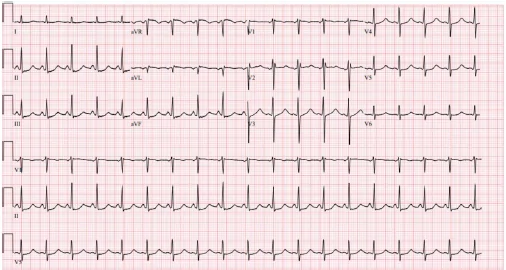
Electrocardiogram after stabilization showing a sinus rhythm with a rate of 111 beats per minute, QRS duration of 89 milliseconds, and resolution of prominent R wave in aVR.

In the setting of recurrent cardiac arrest and severe cardiovascular instability with a history of cocaine ingestion, IV lipid emulsion therapy was initiated. During the initial 20% IV lipid emulsion bolus (1/5 mL per kilogram (kg) of ideal body weight given over 1 minute), the patient briefly lost pulses again. He was stabilized following a third bolus of 50 mEq of IV sodium bicarbonate, 1 mg of IV epinephrine (0.1 mg/mL), and the initiation of the post-bolus 20% IV lipid emulsion infusion (0.25 mL/kg/minute for 1 hour). Image 2 shows the subsequent ECG after emergent stabilization.

Pertinent lab findings revealed an unremarkable complete blood count, lactate of 27.0 millimoles per liter (mmol/L) (reference range: 0.5–1.6 mmol/L), and pH of 6.79 (7.35–7.45). Blood chemistry was as follows: sodium, 139 mmol/L (130–143 mmol/L); potassium, 5.6 mmol/L (3.2–4.8 mmol/L); anion gap, 24 (3–11); creatine, 1.25 mg per deciliter (dL) (0.5–1.5 mg/dL), and phosphorus, 8.4 mg/dL (2.5–5.0 mg/dL). However, the laboratory noted that significant lipemia might have affected the results. Blood ethanol level was 35 mg/dL (< 10 mg/dL), and urine drug screen was positive for cocaine, ethanol, and tetrahydrocannabinol.

The patient was admitted to the intensive care unit. His hospital course was complicated by a brief period of hemodialysis due to anuric renal failure, but he eventually made a full recovery. He was discharged home independently after several weeks of hospitalization.

*CPC-EM Capsule*

What do we already know about this clinical entity?

*Intravenous (IV) lipid emulsion therapy serves as a “lipid sink” to treat cardiac instability caused by lipophilic drug toxicity, reducing free drug bioavailability.*

What makes this presentation of disease reportable?

*Use of IV lipid emulsion therapy contributed to a neurologically intact outcome in a patient presenting with cardiac arrest due to cocaine toxicity.*

What is the major learning point?

*Intravenous lipid emulsion is an available adjunct for treating cocaine-induced cardiac complications, such as bradycardia and circulatory collapse.*

How might this improve emergency medicine practice?

*Clinicians could consider IV lipid emulsion therapy in the resuscitation of critically ill patients experiencing cocaine overdose.*

## DISCUSSION

Intravenous lipid emulsion therapy has served various clinical purposes since its introduction in 1962, ranging from propofol formulation to total parenteral nutrition compounding. However, it emerged as a treatment for lipophilic overdoses in the late 1990s.^3^,^4^ While IV lipid emulsion is known for its effectiveness in management of local anesthetic systemic toxicity, it has additionally been reported as effective in treating overdoses of antipsychotics, antidepressants, antidysrhythmics, calcium channel blockers, and cocaine.^5^–^8^ The precise mechanism of action of IV lipid emulsion is not entirely understood, although the lipid sink theory remains the leading hypothesis. This theory proposes that introducing a volume of lipids provides an alternative binding surface for lipophilic substances, thereby sequestering them from their active states.^2^

Cocaine is a highly lipophilic substance and a potent, dose-dependent sodium channel blocker.^9^ The resulting sodium channel blockade, combined with a concomitant catecholamine surge, can lead to life-threatening arrhythmias—particularly in cases of massive ingestion—as demonstrated in this case. Other case reports have documented the successful use of IV lipid emulsion in patients exhibiting cardiovascular instability following cocaine use; however, these presentations were typically marked by tachyarrhythmias, in contrast to the bradycardia observed in our patient.^6^–^8^

Although it did not delay the administration of IV lipid emulsion, bedside discussion occurred regarding the patient’s bradycardia, given that cocaine classically induces tachycardia. Consideration was given to the possibility of polysubstance overdose or the ingestion of a different agent, such as a beta-blocker, which more typically causes bradycardia. The patient’s family remained adamant that only cocaine and a small amount of alcohol had been ingested prior to the cardiac arrest. A comprehensive serum drug screen was ordered but not completed until late in the hospital course, at which point acute ingestants from the initial presentation were no longer detectable.

A literature review revealed few case reports of bradycardia associated with cocaine use; however, those reports are largely limited to discussion of baseline bradycardia as a marker of chronic use rather than acute intoxication.^10^–^12^ The sodium channel blocking effects of cocaine may explain this finding supported by the prolonged QRS and prominent R wave in aVR.^13^ As a class 1 sodium channel blocker, cocaine can slow cardiac conduction by decreasing both the slope and amplitude of phase 0 of the myocyte action potential.^14^ Further, sodium channel blockade predominates over sympathomimetic stimulation in high cocaine doses producing bradycardia and wide complex dysrhythmia.^15^ Additionally, acidosis and concurrent alcohol use (producing cocaethylene) can intensify the sodium channel blockade.^15^

## CONCLUSION

Management of cocaine-associated cardiac arrest presents many challenges due to its multiple toxic effects on the cardiovascular system, particularly in the setting of massive ingestion. Intravenous lipid emulsion therapy can be used as a lipid sink to help stabilize these patients, especially in the setting of wide complex dysrhythmia or cardiovascular instability not responding to typical interventions.
